# Dr. Clark on Tubercular Phthisis: (From the Cyclopœdia of Practical Medicine)

**Published:** 1835-04-01

**Authors:** 


Dr. Clark *on Tubercular Phthisis:* (from the *Cyclopædia of Practical Medicine.*)

By the gradual progress of the physical and mechanical sciences, and particularly by the more extended study of natural history, medical writers, during the present century, have observed facts and deduced inferences with more success than at any former period in the history of our art. Fine-spun theories, which ingeniously accounted for every thing, and too often elevated exceptions into rules, have been almost universally abandoned; and, instead of disputing about proximate causes, or the vital principle, the medical philosophers of the present age have directed all their attention to the facts presented to their observation, whether they refer to the causes and symptoms of diseases, or to the action of remedial agents.

The Treatise of Dr. Clark now before us affords an excellent illustration of the above remarks. Unlike most writers on this subject, who either content themselves with giving a long and tedious detail of the patient's sufferings, from their supposed origin in the first catarrh, to the last fatal diarrhœa, or pneumonia, which lays him in his grave; or who amuse their readers with various theories founded on a very limited experience; he exposes, in a short introductory section, his own more extended views of the disease, and then gives, in the second section, a clear exposition of the *tuberculous constitution* and *tuberculous cachexia,* containing a description of the constitutional signs by which a disposition to phthisis pulmonalis is characterized.

The third section begins with an account of the more common or general forms of phthisis. The symptoms by which the actual existence of tubercles in the lungs may be detected in the very earliest stages, are here pointed out with great clearness.

The average duration of the disease is illustrated by two tables, in which the facts collected by Messrs. Bayle and Louis are so arranged, as to render evident the conclusions to which they lead.

The various circumstances which modify the regular course of the disease are then treated at full length, under the heads of *Acute Phthisis, Chronic Phthisis, Phthisis in Infancy and Childhood, Febrile Phthisis,* and *Latent Phthisis.*

Much new and highly interesting matter will be found under each of these heads, and we fully agree with our author, "that such distinctions are not mere pretensions to refinement, but, on the contrary, are of great utility, both as regards the diagnosis and the treatment of the disease; for, as we shall find, each of these forms has something in its character, which it is important to mark, in order to distinguish the nature of the disease at an early period of its course." (P. 12.)

An attentive study of this section will save the practitioner the mortification of occasionally mistaking this fatal disease for a simple catarrh in some cases, and for dyspepsia or fever in others; thus depriving his patient of the only chance of safety, which a correct diagnosis might have afforded: indeed this section, together with section 4th, which is devoted to the consideration of particular symptoms, the physical signs and diagnosis, constitutes one of the most instructive parts of the whole work. Every portion bears marks of the deep study and patient observation of the author, and abounds with those useful and sententious remarks, and that copious detail, which distinguish the knowledge acquired at the bedside from that merely derived from books. We remark particularly the accuracy with which the close relation of the symptoms and physical signs to the morbid changes throughout the course of the disease is expressed, and how correctly they are appreciated and separated from those of other diseases, in forming the diagnosis. In giving a clear and succinct description of the information to be derived by observing the respiratory movements, and by the employment of auscultation and percussion, the author concludes with the following paragraph.

"By adopting this plan of careful investigation on being first consulted, we do not hesitate to express our conviction that the greater number of cases of tuberculous phthisis would be discovered at a much earlier period of their course,—often, we are satisfied, many months, nay even occasionally years, before they now are, from the careless manner in which this class of patients are too commonly examined. Until we adopt a more minute and methodical system of inquiring into the history of the case, and, in addition to the usual symptoms of pulmonary disease, avail ourselves of the light afforded by auscultation in the most extended sense of that term, tubercular disease of the lungs can rarely be detected at such an early period of its progress as will give reason to hope that its further advancement may be prevented. In the present superficial mode of conducting our inquiry into the nature of such causes, the disease of the lungs has often made considerable progress, when the patient is said to be merely threatened with it; and tracheal or bronchial irritation are the terms employed to account for symptoms which a close investigation would trace to a deeper source. We must not be satisfied with a few rough and slovenly thumps on the upper part of the chest, or even with the use of the ear or stethoscope for a few seconds, applied as if he were afraid rather than desirous of ascertaining the real conditions of the lungs. Such superficial examinations, if they deserve the name, are worse than useless: with semblance of doing something, they really expect nothing, unless it be to deceive the patient and his friends, and bring this method of diagnosis into unmerited disrepute. Nature will not be interrogated in this rude manner: her operations must be observed with care, and studied with attention, before we can hope to interpret them with fidelity and precision." (P. 31.)

The fifth section, on the morbid anatomy of phthisis, presents nothing with which the labours of the French pathologists, and our indefatigable countrymen, Starke and Carswell, had not made us acquainted.

Section sixth contains an account of the principal complications of phthisis.

1st. Diseases of the organs of respiration, including ulceration of the epiglottis, larynx, and trachea, perforation and other affections of the pleura. 2d. Diseases of the abdominal viscera, including morbid conditions of the mucous membrane of the stomach, enlargement of that organ, ulceration of the intestines, fatty degeneration of the liver, &c. Much information, highly interesting to those especially who are unacquainted with the labours of Louis and other French pathologists, will be found under these heads.

Section seventh comprises the statistical history of phthisis, arranged under "prevalence of tuberculous diseases at the different periods of life; influence of sex, of certain occupations, and of climate, in producing phthisis." All these questions are illustrated by tables which render our knowledge more complete and certain than that which could be derived from the experience, however extensive, of an individual. Very much, however, yet remains to be done on this subject, both as regards the collection and tabular arrangement of facts, and their subsequent reduction to general laws, before a full dependence can be placed on the accuracy of the conclusions drawn from them. When speaking of the influence of occupation, our author, in treating of the effects of irritating matters applied to the lungs, mentions the theories of Dr. Alison, and some other pathologists, who believe that inflammation, arising from such sources, is a frequent cause of tubercles, and gives it as his opinion that, although the symptoms attending the disease produced by the causes have a great affinity to those of phthisis, yet, in the majority of cases, the appearances found on dissection differ materially from those of tuberculous disease, and that the inhalation of such substances can prove an exciting cause of tubercles only in persons of a tubercular constitution.

Section eighth, *on Tuberculous Disease in Animals,* contains an enumeration of the mammalia and birds in which it has been found. The author believes that no animal is exempt from the liability to it, when subjected to the exciting causes. He even adduces some observations by Mr. Newport respecting the presence of a substance resembling tuberculous matter in certain insects.

The three sections which follow, on the *causes, nature,* and *prevention* of the scrofulous diathesis, involve questions of the highest importance, not only to the medical practitioner, but also to the public at large.

It is much to be lamented that the peculiar conformation of mind which enables those possessing it both to observe at once particular facts with attention, and to form general laws by the use of their reason, should so rarely exist. The circumstance that men are generally either matter-of-fact people or theorists has tended more than aught else to retard the progress of science. One man gathers his facts with the greatest possible industry, never thinking of their combinations or arrangement, till his mind resembles the museum of the slovenly virtuoso, where specimens of all that exists in the three kingdoms of nature are jumbled together: another engrossed in contemplating the varied forms and combinations, which his ill-regulated and over-excited imagination gives to a number of facts imperfectly observed, mistakes the vagaries of his fancy for the truth of nature, and instinctively, and almost unconsciously, shuts his eyes to the study of facts which might serve to throw doubts over what he has made so clear.

In the instance before us, it must be allowed that the generalizations of the medical philosopher are even more useful than the minute researches of the most accurate pathologist; for a perfect knowledge of the condition of the lungs, and the symptoms which arise in the course of the disease, are both subjects of very secondary importance, when compared with the study of those causes which produce the scrofulous diathesis, and of the means by which it may be removed, or the deposition of its products prevented. We may be able to detect the smallest vomica,—we may have examined hundreds of tuberculous subjects,—and we may employ all the balsams, astringents, and fumigations, recommended for ulcer of the lungs by the ancients, with all of iodine and chlorine inhalations that have been added to the vast farrago of phthisical remedies by the moderns; we may diet our poor patient, or feed him on beef-steaks and porter; still the grand result will remain the same: society will have to lament the loss of almost an equal number of her members. We ought not, of course, to be ignorant of the subjects just mentioned: on the contrary, it is only by our perfect acquaintance with them that we can hope to gain the confidence of our patients, and the respect of our professional brethren. The enlightened medical practitioner, however, feels that his duty is but half done when he can only detect organic disease, and mitigate symptoms: the grand object of his ambition ought to be the prevention of disease, the diminution of the general mortality, and the extension of the mean duration of human life. In relation to these questions, the immense importance of increased attention to this disease has never till now been brought before the public, with the force it deserves. Organic diseases, depending on a modification of the whole economy, are, with a few exceptions, developed slowly; and are almost constantly produced by the action of external causes, which may be detected by a little attention, and then very generally avoided or removed. Whoever considers this with due attention, will not attempt to deny that tubercular disease, which now carries off, in one form or other, more than one sixth of the inhabitants of Europe, before they reach the prime of life, might become comparatively a rare disease.

But, before we can hope to see this most desirable end accomplished, many changes must take place in the very commonest usages of our lives; for the disease is so extended, and the causes which produce it so various, and so interwoven with our customs and habits of life, that no great diminution can be effected till the science of hygiene is more sedulously cultivated, and its principles more widely diffused; in fact, not till the attention of the public has been fully awakened and directed to these points for a great number of years. The man who could convince the world of the possibility of preventing the development of tuberculous disease, by efforts which should begin before the formation of the fœtus, follow the infant through childhood and youth, as our author has done, and, for the sake of posterity, relax not till the last hour of existence, would render a greater service to mankind than even the immortal Jenner. But it is to be feared we shall long hope in vain: the bad effects of neglect in this instance follow the causes at too great an interval, and the physician will therefore find as much reason as the moralist, to lament the innate propensity of man to prefer present ease and pleasure to future health and happiness. With all the highly-boasted diffusion of knowledge in the present day, it is surprising to find the means of preserving health so entirely neglected. We are much afraid that a great portion of the reproach must be laid on the medical profession: had its members instructed the public as they ought, the care for posterity would have extended beyond the vain ambition of transmitting to it wealth and honours, which, without health, are but a gorgeous mockery. We hope that a new era is approaching, and that these subjects will be studied as they deserve.

Our author begins his account of the causes of tuberculous disease by considering the influence of hereditary transmission, and takes great pains to show that a disposition to tubercles may be communicated to the offspring, not only when the parents actually labour under the disease, but also that various other morbid states of the parent may have this effect upon the offspring. Of these, disease of the digestive organs is the most powerful.

"Of all diseases, we consider dyspepsia the most fertile source of cachexia of every form; for this plain reason, that a healthy condition of the digestive organs, and a proper performance of their functions, are essential to the due preparation of the food, and consequently to the supply of healthy nourishment to the body. The adjusting powers of the system may do much to correct a disordered condition of the different functions concerned in the process of assimilation, by means of the increased activity of the healthy organs; but the system cannot long continue in a healthy state when any one important function connected with nutrition is materially deranged. Without, however, entering into this most interesting subject, we consider it an established fact, (it is so at least to us,) that dyspepsia, and any other disease which induces a cachectic state of the parent, shows itself either in the tuberculous constitution of the children, or in their strong tendency to become the subjects of those disorders which generate such a constitution,—such as that form of dyspepsia which has been denominated *strumous* by Dr. Todd."* (P. 53.)

Of all the forms of hereditary transmission, the author considers as the most common, though the least generally understood, that where the child is merely predisposed to

* See Cyclopædia of Practical Medicine, article *Indigestion*, for a masterly account of dyspepsia under its various forms.

functional derangement, capable of generating the tuberculous constitution.

The injurious effects of *deficient exercise, excessive labour, improper clothing, want of cleanliness, abuse of spirituous liquors, mental causes*, and, lastly, the question of *contagion*, are treated in succession. The author concludes this subject with the following summary.

"Reviewing what has been said respecting the causes of tubercular cachexia, they may be stated generally to comprehend all those circumstances which debilitate and increase the irritability of the system, impede the due digestion and assimilation of the food, diminish the various secretions and excretions, and induce internal sanguineous congestion. Defective assimilation, from whatever cause it proceeds,—whichever be the first link in the chain of morbid processes,—induces, according to our view, tuberculous cachexia; and whether the primary error exist in the inadequate supply of food, or in the incapacity of the organs to extract from this the elements of nutrition, to assimilate and apply them to the reparation, growth, and various purposes of the animal economy, the ultimate result will be the same." (P. 56.)

The causes which determine tuberculous disease in the lungs are next described. They are divided into two classes: 1st, those which act immediately on the organs, as bronchitis, pneumonia, hæmoptysis, and some other pulmonary affections. Bronchitis and pneumonia have some effect as exciting causes, only when the constitutional diathesis preexists. The second class of causes act partly on the lungs, and partly on the general system: such are fevers, and more particularly those of the eruptive kind, as rubeola, scarlatina, and variola.

In the tenth section we have an excellent description of the *pathology of phthisis*, and of tuberculous diseases in general, in which the author gives a minute account of his opinions respecting the constitutional nature of the disease.

"In whatever point of view we may regard tuberculous cachexia, we shall find its phenomena explicable only by admitting that it depends on a general modification of the whole and every part of the animal economy; and that all notions which regard it as the morbid degeneration of any organ or tissue, or of any particular system, or the morbid modification of any single fluid, are necessarily erroneous, and founded on limited views of its nature and laws, and totally inadequate to explain its phenomena." (P. 60.)

Our author remarks, that the remote causes of phthisis, however variously they may operate, do so by inducing some positive pathological conditions, which, being constantly present wherever tuberculous disease is formed, may be regarded as necessary to its production; that, in the actual state of physiology and pathology, all these conditions cannot with certainty be defined; still that it would not be difficult to point out some of the more important links of the chain which connects special functional disorder with the formation of tuberculous cachexia; and he refers to a "congestive state of the venous system of the abdomen," as constituting one of the most obvious and important of these pathological conditions in a practical point of view.

His views are the result of practical observation, that a disposition to this congestive state of the abdominal circulation constantly exists in strumous children; that it is, besides, a precursor of tubercular cachexia; and that it presents itself more under the form of dyspepsia, and its concomitants, in the middle period of life, often obscuring the pulmonary affection: tubercular phthisis rarely occurring at this age without this complication. Our author refers to the authority of many German physicians of the last century, to the opinions of Kæmpf and his disciples, and to the observation of Portal, on this subject, with whose labours he was not acquainted till he had himself, from his own experience, arrived at the same conclusions.

"Abdominal plethora," our author says, "when once established, gives rise to a series of deranged functions in the digestive organs, the lungs, and the skin, which, by impeding digestion and assimilation, affect the whole animal economy. These are manifested in imperfect biliary secretion, constipated bowels, and irritated mucous surfaces, in congestion of the lungs, and a dry and harsh state of the skin.

"In consequence of the overloaded condition of the venous system, the heart, generally feeble in the tuberculous constitution, is oppressed, and the arterial circulation impeded and enfeebled. In this state of the system, very slight exciting causes induce disease, especially inflammation and hemorrhage: hence arises the constant liability of strumous subjects to inflammatory diseases of a subacute or chronic character; and hence also we derive an explanation of the hemorrhage to which they are peculiarly liable, even at a very early stage. The same pathological state of the abdominal circulation forms the remote cause of the various congestive and chronic diseases, so common in the strumous subject; such as glandular swellings, cutaneous eruptions, &c."

Section eleventh contains a very comprehensive view of the precautions to be adopted in attempting to prevent the occurrence of tuberculous diseases: 1st, as regards the parents; 2d, as regards the offspring.

Under the first head, the question relating to marriage and the health of the parents is noticed, and the rules to be observed by delicate females during pregnancy are laid down with much judgment. But it is under the second head, in which the prevention of the disease in infancy, childhood, and youth, is treated of separately, that the author displays, in its fullest extent, that power of observation, and that practical application of facts, by which his writings are characterized.

We recommend strongly the study of the hygienic remarks in this section to our professional brethren: indeed, we think that every parent ought to be acquainted with the excellent rules laid down on nursing, dress, bathing, air, exercise, and education.

As the tubercular cachexia and phthisis so often occur about the period of puberty, the author under this head gives an account of the remedies for the cure of this morbid state of the constitution, and for the prevention of phthisis. "The utility, however, of these remedies is not confined to this period of life: they may be employed at any age, almost from infancy, when the circumstances of the patient indicate their use; and some of them, although ranked among preventives, are often applicable in the early stages of phthisis." He divides them into *alteratives*, *purgatives*, *tonics*, *bathing*, &c.

Of the first class, mercury will be found a very valuable remedy, when the secreting functions of the liver are disordered; but if, on the other hand, it be carried beyond its alterative effects on the hepatic system, it seldom fails to prove injurious. Taraxacum is considered "a very valuable remedy, from its power of diminishing abdominal plethora, and its special influence on the urinary and biliary secretions." The opinions of several distinguished German writers are quoted in its favour, and good practical hints are given as to the best mode and time of administering it. He has found sulphureous waters a valuable remedy, when the strumous constitution is complicated with cutaneous affections, especially "when the skin is coarse and dry, and the whole constitution of a torpid character; but, in the more delicate class of strumous patients, with a thin skin, the use of sulphureous waters requires much caution."

Mineral waters, the author is of opinion, are superior to all other alterative remedies. "The operation of these invaluable remedies may be so directed as to promote all the secretions and excretions, to influence the functions of almost every organ, and improve the condition of the circulating fluids."

The cautions to be observed in the administration of purgatives, and the circumstances under which the other means included in this section may prove useful, are pointed out with great minuteness.

Of the general remedies, venesection is first considered, and the peculiar practice of small and frequent bleedings, adopted by Dover and others, is discussed. The author believes that general bleeding would seldom require to be repeated, if proper attention be paid to the regimen, and to the diminution of the abdominal plethora; and that local bleeding, by cupping on the chest, is the most effectual way of relieving the inflammatory action accompanying tubercles, after one or more general bleedings have diminished the congestion.

Our author insists strongly on the efficacy of emetics, and numerous authorities are quoted in their support. He thinks their action may be accounted for, on rational principles, by the new views of Dr. Carswell* respecting the seat of tubercle, and earnestly recommends their use, as holding out "the rational hope of being made one of the most efficient means of preventing the localization of tuberculous disease in the lungs in many cases, and perhaps removing it in some others."

The advantages to be derived from the use of digitalis and iodine are discussed; after which follow some excellent observations on the climate most suited to the various forms and stages of the disease. The valuable labours of our author on this subject are already too well known, and too highly appreciated, to require any mention in this place.

The employment of local remedies, consisting of local bleeding, counter-irritants, inhalation of tar-vapour, iodine, and chlorine, is then considered. After remarking the partial nature of these agents, the author concludes the subject of local remedies with the following observations, in the truth of which we perfectly acquiesce.

"When more rational and just views of the pathology of phthisis are generally entertained by the profession, we shall cease to hear it asserted that this disease is to be cured by local applications. We do not, however, condemn such measures as useless:

* Illustrations of Elementary Forms of Disease. Fasc. 1, and art. Tubercle. (Cyclopædia of Practical Medicine.)

on the contrary, we consider them valuable as palliatives, and of great service as adjuncts to those remedies which are directed to amend the condition of the general health, and to correct the tuberculous diathesis; but we certainly disapprove of any local remedy being relied on as the principal means of curing a disease which depends upon a morbid state of the constitution: such an error is founded on imperfect views of the real nature of tubercular phthisis, is productive of much mischief in practice, and cannot be too strongly reprobated." (P. 84.)

Some admirable practical observations then follow, on the treatment of particular symptoms; and the author concludes with the treatment of the advanced stages of the disease.

The very important nature of these remarks, and the close style in which they are written, preclude the possibility of condensation, though we are sorry to leave the author with this imperfect notice of his excellent work. We hope that a knowledge of its contents will not be confined to the purchasers, however numerous, of the valuable book in which it forms one of the best articles; for we have seldom seen a medical work more deserving of general circulation, or one that we would more zealously recommend to the younger members of the profession, whether we regard its clear and simple language, (a point much neglected by professional writers,) or the paramount importance of the subject, and the enlightened views of the author.

